# Prevalence of Toxoplasma gondii Infection Among Blood Donors: A Systematic Review and Meta-Analysis

**DOI:** 10.7759/cureus.85403

**Published:** 2025-06-05

**Authors:** Maria Kantzanou, Evangelos Kostares, Georgia Kostare, Evangelia Papagiannopoulou, Michael Kostares, Athanasios Tsakris

**Affiliations:** 1 Microbiology, National and Kapodistrian University of Athens School of Medicine, Athens, GRC; 2 Anatomy, National and Kapodistrian University of Athens School of Medicine, Athens, GRC

**Keywords:** blood donors, meta-analysis, molecular analysis, prevalence, serological analysis, toxoplasma gondii

## Abstract

Our study aims to thoroughly evaluate the prevalence of *Toxoplasma gondii *infection among blood donors, utilizing robust methodologies to inform public health policies and improve transfusion safety measures. An extensive literature search was executed across databases such as Medline, Scopus, and Cochrane Central, identifying observational studies reporting *T. gondii* seroreactivity and detection prevalence in blood donors across various regions. Heterogeneity was assessed visually via forest plots and statistically using Cochran’s Q test (with p-values) and the I² statistic with 95% CIs. The meta-analysis estimated pooled prevalence rates with a 95% CI and included rigorous quality assessments, outlier, and influential analyses to verify the findings’ validity. Thirty-eight studies, published between 1998 and 2024, were incorporated into our review, revealing a *T. gondii* reactivity prevalence of 35.7% (95% CI: 29.8-42%) through serological diagnostic methods and 1.9% (95% CI: 0.2-4.9%) through molecular methods. The substantial heterogeneity detected across studies underscores the necessity for future investigations to explore the determinants of *T. gondii* reactivity and detection prevalence, addressing the review’s identified limitations with more targeted research designs.

## Introduction and background

*Toxoplasma gondii *is an intracellular parasitic protozoan and is classified under the phylum Apicomplexa and the subclass Coccidia. This microorganism exists in three main infectious stages: the oocyst, the tachyzoite, and the tissue cyst. Human infection with *T. gondii *can occur through various means, including foodborne transmission, animal-to-human transmission, congenital infection, organ transplantation, and blood transfusion [1-3].

One of the main sources of *T. gondii *transmission includes the consumption of undercooked or raw meat from infected animals, such as pork, lamb, and venison, which may harbor tissue cysts, and the ingestion of shellfish like oysters, clams, and mussels that can be contaminated with oocysts from polluted water. It is important to note that direct contact with animals does not typically result in transmission. Transmission can occur through the consumption of undercooked or raw meat containing tissue cysts or the ingestion of food or water contaminated with oocysts shed by infected cats. While direct contact with infected animals is not a significant route of transmission, handling contaminated surfaces or consuming unwashed produce can pose a risk. To minimize the risk of infection, proper food preparation, thorough washing of fruits and vegetables, good hygiene practices, and avoidance of potentially contaminated water sources are essential [1,4].

Transplant patients receiving organs from donors who are already positive for *T. gondii *antibodies, while the recipients are negative, are at risk of contracting the disease. The typical mode of toxoplasmosis development in bone marrow, hematopoietic stem cell, liver transplant patients, and those with AIDS is the reactivation of latent infections in the recipient. During gestation, if a pregnant woman acquires *T. gondii *for the first time, the parasite can reach the fetal circulation by infecting the placenta, posing a risk to the developing baby [1,4,5]. Furthermore, *T. gondii *can be transmitted through blood or white blood cells from both individuals with normal immune function and those with compromised immune systems. Infections in laboratory staff have occurred through accidental contact with contaminated needles or exposure to biological materials, such as tissues or fluids from infected animals, during laboratory procedures [1,5,6].

In the United States, approximately 11% of individuals are estimated to have contracted *T. gondii *[7]. Studies conducted in various regions worldwide have revealed infection rates exceeding 60% in certain populations for *T. gondii*. Recently, a comprehensive meta-analysis conducted by Wang et al. [8], which encompassed 74 studies (including 25,898 HIV-infected individuals), determined the prevalence of *T. gondii* reactivity to be 35.8% (95% CI: 30.8-40.7%). Infection rates tend to be highest in regions characterized by hot, humid climates and lower altitudes, as these environmental conditions favor the survival of oocysts [1]. While often asymptomatic in immunocompetent individuals, toxoplasmosis can cause severe disease in immunocompromised patients, including transplant recipients and individuals living with HIV [1,9].

Blood donors represent a subgroup for *T. gondii *surveillance because their donations are intended for transfusion into recipients who may be immunosuppressed, such as neonates, cancer patients, or organ transplant recipients. Even when asymptomatic, seropositive donors can harbor latent parasites, and rare but documented cases of transfusion-transmitted toxoplasmosis raise concerns about blood safety [9].

There is an abundance of variation in the prevalence of *T. gondii *reactivity and detection among blood donors, according to scientific literature. Our systematic review and meta-analysis seek to close this information gap by offering a thorough evaluation of the prevalence of *Toxoplasma* species in this particular group. Our research intends to provide important insights to the scientific community by revealing the full magnitude of this hidden reactivity within the blood donor population by combining data from other investigations.

## Review

Materials and methods

Search Strategy

**Figure 1:**
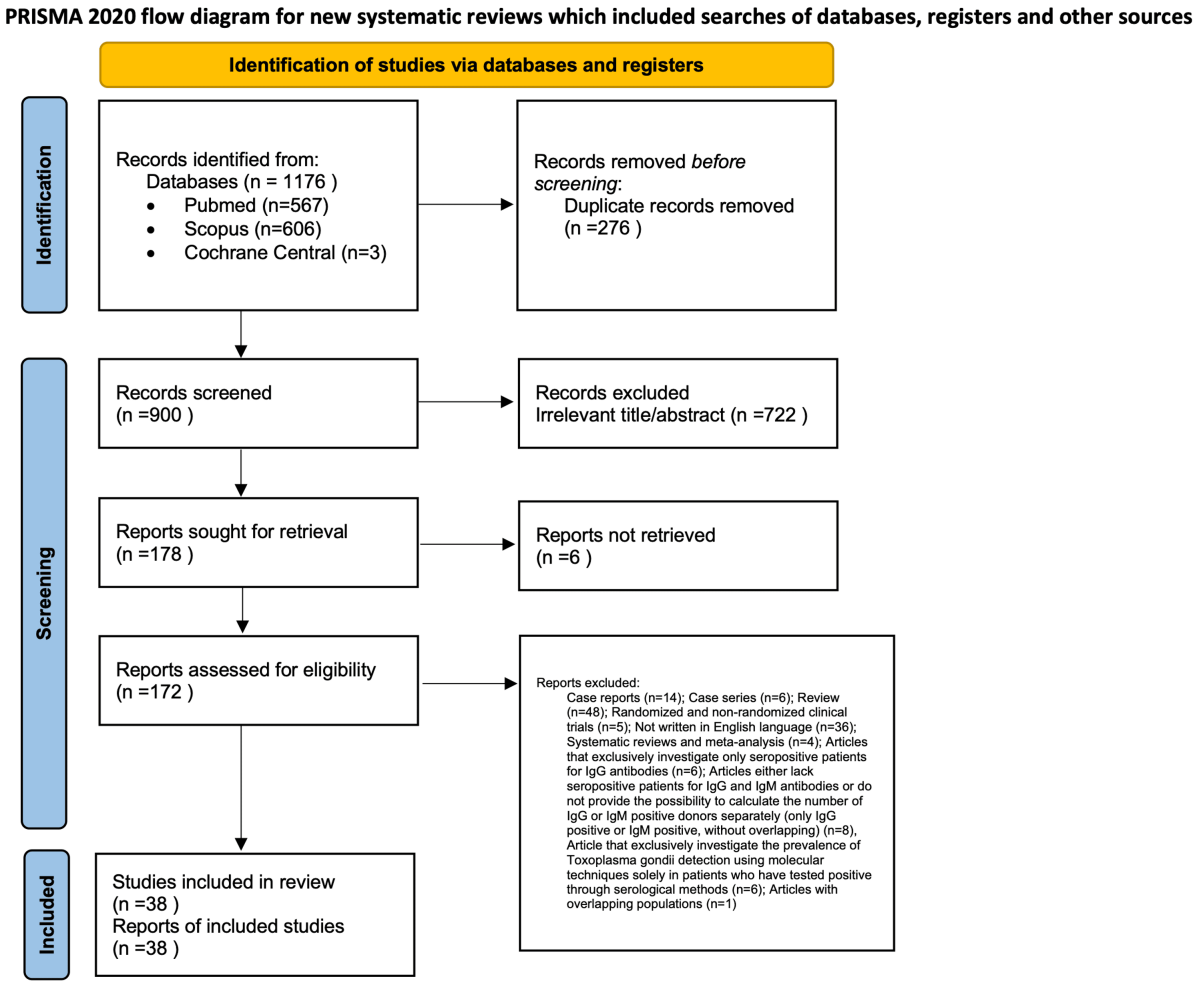
Flowchart depicting the systematic search results from the relevant studies’ identification and selection PRISMA, Preferred Reporting Items for Systematic reviews and Meta-Analyses

A comprehensive search of the literature was done in compliance with the Cochrane Handbook for Systematic Reviews of Interventions. This systematic review was organized and reported in accordance with the Preferred Reporting Items for Systematic reviews and Meta-Analyses (PRISMA) guidelines [10]. The PRISMA checklist, available in Appendix A, was utilized to facilitate the systematic review process. A comprehensive literature search was conducted, covering works from their inception up to January 9, 2025, using the Medline/PMC Central (via PubMed), Scopus, and Cochrane Central databases, as shown in Figure 1. Two reviewers independently carried out the search, using a combination of keywords such as “toxoplasmosis”, “toxoplasma gondii”, “blood donors”, “blood transfusion”, “blood bank”, “blood donation”, “prevalence”, “incidence”, and “rate”. The detailed search strategy for each database is provided in Appendix B. To ensure no relevant studies were missed, we also thoroughly reviewed the reference lists of the identified studies. We used Zotero reference management software (version 6.0.18) to organize and manage the collected research meticulously [11]. Duplicate references were carefully removed to ensure the dataset’s integrity. Two independent reviewers then scrutinized the remaining articles individually. The study selection process was conducted in two phases: first, titles and abstracts were screened to exclude studies that did not meet the predefined inclusion criteria, followed by a thorough evaluation of the full texts of the remaining papers. Any disagreements were resolved by consensus among team members, ensuring consistency in decision-making. This systematic approach ensured the compilation of a comprehensive and reliable set of studies for analysis.

Criteria for Study Selection and Data Extraction

Following an exhaustive and meticulous search across multiple databases, we meticulously defined our eligibility criteria using the PECOS framework. This rigorous process was undertaken to ensure maximum clarity, precision, and thoroughness in our systematic review and meta-analysis, which centers on the prevalence of *T. gondii* reactivity and detection among blood donors. By adhering to this structured approach, we aimed to achieve a comprehensive and reliable synthesis of the available data. In Table 1, the PECOS framework is presented.

**Table 1:** PECOS criteria

PECOS criteria	
Population (P)	Blood donors. With the aim of collecting information from a wide range of participants in various places and times, the study focuses on determining the prevalence of *Toxoplasma gondii *reactivity or detection among blood donors.
Exposure (E)	The exposure under investigation is the presence of *T. gondii *reactivity or *T. gondii *detection in blood donors.
Comparison (C)	Since our goal was to measure the prevalence of *T. gondii *reactivity and detection in blood donors, the study’s design did not allow for a direct comparison component.
Outcomes (O)	The prevalence rate of *T. gondii *reactivity and detection among blood donors, as determined by serological and molecular diagnostic techniques, is the study’s main goal.
Study types (S)	Our inclusion criteria encompassed solely observational studies, including cohort, case-control, and cross-sectional studies.

Table 2 provides a clear framework for the selection of studies by outlining the specific inclusion and exclusion criteria for the systematic review and meta-analysis.

**Table 2:** Inclusion and exclusion criteria

Criteria	Inclusion	Exclusion
Study types	Observational studies, including cohort, case-control, and cross-sectional studies	Case reports, case series with ≤20 participants, review articles, randomized clinical trials, nonrandomized clinical trials, animal studies, letters to the editor, books, expert opinions, conference abstracts, and studies without full-text availability
Language	English	Non-English articles
Publication date	No restrictions	None
Study design	Articles that examined specifically the prevalence rates of *Toxoplasma gondii *reactivity and detection among blood donors were included with no restriction on publication date. Also, it is important to note that when considering the meta-analysis for the molecular method of diagnosis, we include studies that have implemented molecular diagnostic methods in the entire study population, rather than solely focusing on the individuals identified as positive through serological methods. This approach enables a more homogeneous population since a negative result from a serological method does not necessarily indicate a negative result from the molecular method	Articles that exclusively investigate the prevalence of *T. gondii* detection using molecular techniques solely in patients who have tested positive through serological methods, articles investigate only seropositive patients for IgG antibodies [12-14], studies either lack the seropositive patients for both IgG and IgM antibodies or do not provide the possibility to calculate the number of IgG- or IgM-positive donors separately (only IgG-positive or IgM-positive, without overlapping) [15,16] and studies regarding other infections
Population overlap	Most recent or comprehensive publication used if populations overlap	Publications with overlapping populations

A concise summary of the inclusion and exclusion criteria is presented below. Eligible studies were observational in design (cross-sectional, cohort, or case-control) and specifically investigated the prevalence of *T. gondii *reactivity or detection among blood donors using serological (e.g., IgG and IgM) and/or molecular (e.g., PCR) diagnostic methods applied to the full study population. Studies were excluded if they lacked primary data (e.g., reviews and case reports), focused on nonrepresentative subgroups (e.g., only seropositive individuals), had overlapping populations without new data, or did not provide sufficient detail to extract or calculate relevant prevalence measures.

Data Extraction

From each included study, we collected the following details: the authors’ names, year of publication, type of study design, continent and country of origin, duration of the study, total number of blood units, percentage of male participants, average age, number of patients with *T. gondii *reactivity, and the diagnostic procedures used.

Quality Assessment

Using the Quality Assessment Tools from the National Heart, Lung, and Blood Institute (NHLBI), two researchers independently evaluated the quality of their research. This was accomplished by using the NHLBI Observational Cohort and Cross-Sectional Studies Quality Assessment Tool, specifically. Every research study was carefully examined to find any possible methodological or survey implementation problems that would jeopardize the study’s internal validity. In order to complete the assessment, participants had to answer 14 questions with the following options: “yes,” “no,” “cannot determine” (for example, because of ambiguous or inconsistent data), “not reported” (for example, when data was missing), or “not applicable” (for example, when a question was not pertinent to the study in question). According to reference [17], these evaluations produced three different study quality levels: “low,” “moderate,” and “high” risk of bias (Appendix C).

Statistical Analysis

We conducted a thorough statistical analysis for the meta-analysis using the metafor package [18,19]. The overall prevalence and its 95% CIs were estimated using the DerSimonian and Laird random effects model. To address variance instability, the Freeman-Tukey double arcsine transformation was applied for outcomes such as IgM antibodies, combined IgG/IgM antibodies, and molecular diagnostic results [20]. Conversely, the logit transformation was used to evaluate the serological diagnostic techniques and the prevalence of IgG antibodies [21].

Heterogeneity: Heterogeneity among the studies was evaluated both visually, through forest plot inspection, and statistically, using Cochran’s Q statistic along with its p-value. Additionally, the Higgins I² statistic, accompanied by its 95% CI, was employed to quantify the extent of true heterogeneity in effect sizes. The I² values were interpreted as follows: 0-40% indicated not important heterogeneity, 30-60% indicated moderate heterogeneity, 50-90% indicated substantial heterogeneity, and 75-100% indicated considerable heterogeneity.

Sensitivity analysis: To identify and assess potentially influential outliers, we screened for externally studentized residuals with absolute z-values greater than two and performed leave-one-out diagnostics [22]. Unless otherwise noted, statistical significance was set at p = 0.05 (two-tailed).

Publication bias: We assessed publication bias using Egger’s test [23], Begg’s test [24], and funnel plots, which are standard methods for comparing data. These tests operate on the assumption that studies with positive results are more likely to be published than those with negative results. However, in a meta-analysis of proportions, there's no clear agreement on what defines a positive result [25]. Therefore, we evaluated publication bias in this meta-analysis qualitatively.

All analyses were conducted using the RStudio program (version 2022.12.0+353) [18].

Results

Results and Characteristics of the Included Studies

From 1,176 articles initially identified, 38 studies were finally included in this analysis. All articles were published from 1998 to 2024 (conducted from 1992 to 2023). All of them were of cross-sectional design. Most of the studies were carried out in Asia (Iran, Turkey, Malaysia, India, Taiwan, Thailand, and Iraq), followed by Africa (Tunisia, Egypt, Algeria, and Côte d'Ivoire), Europe (Serbia, Czechia, Poland, and Romania), North America (Mexico), and South America (Brazil and Peru). Thirty-seven studies encompassing 19,691 participants were included to investigate the prevalence of *T. gondii *reactivity through serological diagnostic procedures, such as ELISA, direct agglutination assay, and electrochemiluminescence immunoassay. Collective analysis revealed that males made up an average of 80.9% of participants, with a mean age ranging from 22.8 to 39.3 years and a median age of 34 years. Seven studies, involving 3,292 participants, explored the prevalence of *T. gondii *detection using molecular diagnostic techniques such as real-time and conventional PCR. In these studies, males accounted for an average of 74.5% of participants, with a mean age range of 22.8 to 38.1 years and a median age of 34 years. All studies were assessed as being of moderate quality. The descriptive characteristics of them are reported in Table 3.

**Table 3:** Descriptive characteristics of included studies

Authors	Year	Study design	Continent of origin	Country	Study period	Total patients	Proportion of males (%)	Mean age (years)	IgG positive	IgM positive	IgG and IgM positive	Seropositive patients	Molecularly diagnosed patients
Gouda et al. [26]	2024	Cross-sectional	Africa	Egypt	2023	420	97.1	32.4	46	22	1	69	NA
Ibrahem et al. [27]	2024	Cross-sectional	Africa	Egypt	2023	630	96.7	31.9	68	37	2	107	NA
Mohammed et al. [28]	2023	Cross-sectional	Asia	Iraq	2019-2020	82	54.9	36.2	20	0	0	20	NA
Yildiz et al. [29]	2023	Cross-sectional	Asia	Turkey	NA	46	95.7	NA	10	0	1	11	NA
Assoni et al. [30]	2023	Cross-sectional	South America	Brazil	NA	1,729	65.1	34.5	797	3	30	830	NA
Belkacemi et al. [31]	2022	Cross-sectional	Africa	Algeria	2018	103	42.7	31	46	0	0	46	NA
Paraboni et al. [32]	2022	Cross-sectional	South America	Brazil	2020-2021	510	52.2	36.6	223	0	8	231	NA
Lupu et al. [33]	2022	Cross-sectional	Europe	Romania	2018	1347	56.1	33.6	NA	NA	NA	618	NA
Pawełczyk et al. [34]	2022	Cross-sectional	Europe	Poland	2013-2016	168	65.5	NA	49	0	1	50	29
Stopić et al. [35]	2022	Cross-sectional	Europe	Serbia	2017-2018	1,095	50.6	NA	224	NA	0	NA	NA
Yilmaz et al. [36]	2021	Cross-sectional	Asia	Turkey	2015	879	90	34	213	8	12	233	NA
Asfaram et al. [37]	2021	Cross-sectional	Asia	Iran	2017	462	97.2	39.3	150	7	9	166	NA
Díaz-Ginéz and Silva-Díaz [38]	2021	Cross-sectional	South America	Peru	2019	92	76.1	NA	71	0	0	71	NA
Lachkhem et al. [39]	2020	Cross-sectional	Africa	Tunisia	2017-2018	800	79.5	35	352	0	3	355	NA
Nakashima et al. [40]	2020	Cross-sectional	South America	Brazil	NA	750	67.5	NA	335	5	21	361	NA
Hosseini et al. [41]	2020	Cross-sectional	Asia	Iran	2014	400	95.3	35.5	287	2	7	296	7
Jati et al. [42]	2020	Cross-sectional	Asia	Malaysia	2017-2018	56	59	NA	24	0	2	26	0
Manouchehri Naeini et al. [43]	2019	Cross-sectional	Asia	Iran	2017	385	94.8	37.2	146	4	6	156	6
Saki et al. [44]	2019	Cross-sectional	Asia	Iran	2015	380	NA	NA	131	2	11	144	NA
Abd El Wahab et al. [45]	2018	Cross-sectional	Africa	Egypt	2016	276	78.3	29.7	135	2	15	152	NA
Kalantari et al. [46]	2018	Cross-sectional	Asia	Iran	2016-2017	500	97.6	NA	313	NA	3	NA	NA
Moshfe et al. [47]	2018	Cross-sectional	Asia	Iran	2015	285	96.8	37	46	0	2	48	NA
Sadooghian et al. [48]	2017	Cross-sectional	Asia	Iran	2014-2015	491	93.9	36.3	184	8	8	200	NA
Stephen et al. [49]	2017	Cross-sectional	Asia	India	2016	275	93.1	34.8	53	0	1	54	NA
Tappeh et al. [50]	2017	Cross-sectional	Asia	Iran	2013	270	96.7	34	102	0	0	102	NA
Zarean et al. [51]	2017	Cross-sectional	Asia	Iran	2013	500	89.6	29.7	125	16	7	148	NA
Siransy et al. [52]	2016	Cross-sectional	Africa	Côte d'Ivoire	2014	106	86.8	NA	60	4	8	72	NA
El-Sayed et al. [53]	2016	Cross-sectional	Africa	Egypt	2014-2015	300	100	22.8	93	2	8	103	18
Alvarado-Esquivel et al. [54]	2016	Cross-sectional	North America	Mexico	2015	408	81.9	31.8	43	NA	12	NA	NA
Mahmoudvand et al. [55]	2015	Cross-sectional	Asia	Iran	2014	500	91	28.9	144	11	5	160	NA
Sarkari et al. [56]	2014	Cross-sectional	Asia	Iran	2012-2013	1,480	94.3	39.1	182	81	23	286	NA
Shaddel et al. [57]	2014	Cross-sectional	Asia	Iran	2013	250	NA	NA	57	NA	1	58	NA
Zainodini et al. [58]	2014	Cross-sectional	Asia	Iran	NA	200	NA	NA	NA	NA	NA	NA	14
Chiang et al. [59]	2012	Cross-sectional	Asia	Taiwan	2010	1,783	63.2	38.1	161	0	5	166	0
Elhence et al. [60]	2010	Cross-sectional	Asia	India	NA	493	94.3	NA	240	9	16	265	NA
Alvarado-Esquivel et al. [61]	2007	Cross-sectional	North America	Mexico	2006	432	86.6	30.4	24	NA	8	NA	NA
Pinlaor et al. [62]	2000	Cross-sectional	Asia	Thailand	1997	345	67	31.4	NA	NA	NA	28	NA
Svobodová and Literák [63]	1998	Cross-sectional	Europe	Czechia	1992-1993	663	83.9	NA	203	6	10	219	NA

Prevalence of T. gondii Reactivity and Detection Among Blood Donors

**Figure 2:**
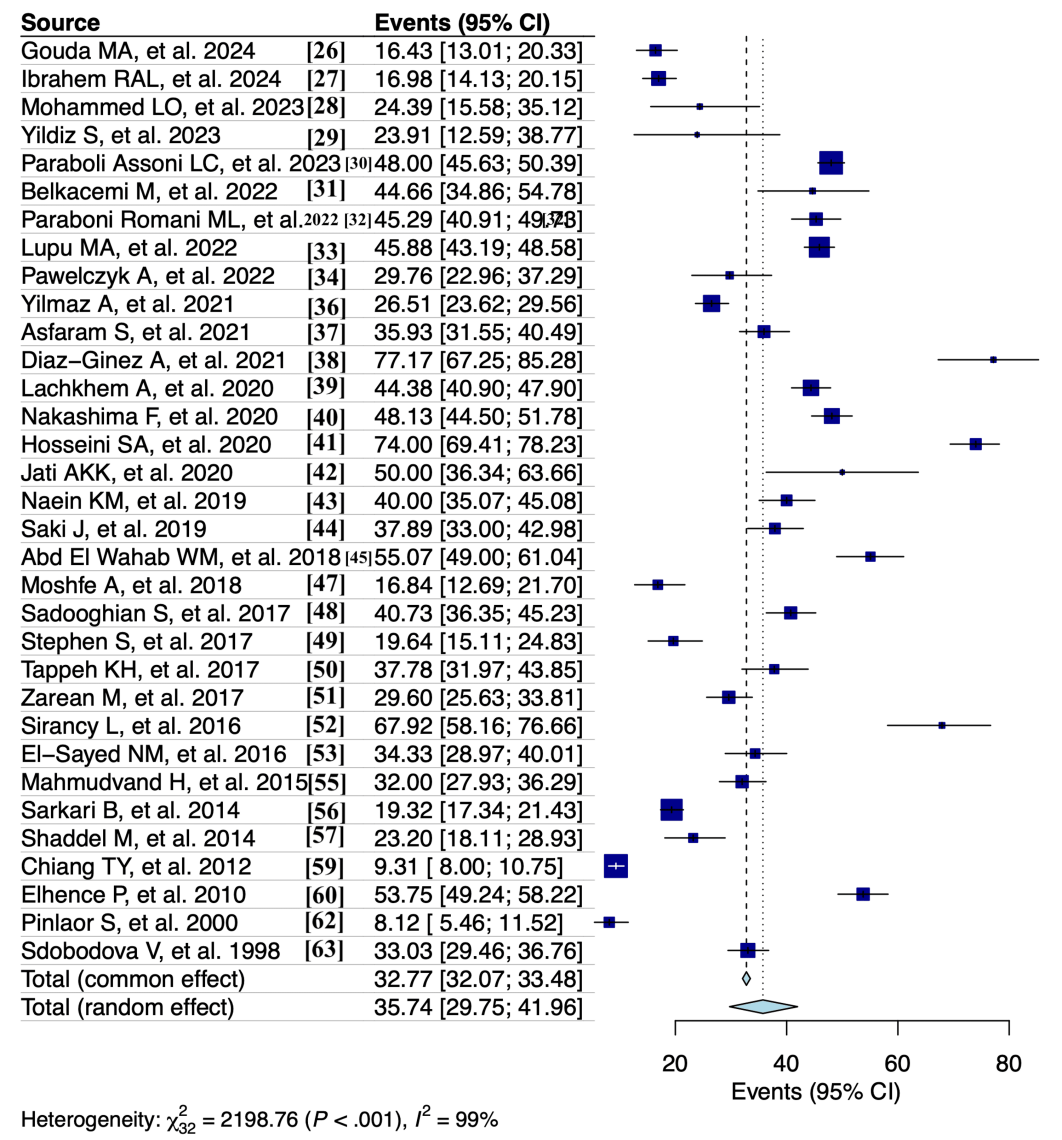
Forest plot showing Toxoplasma gondii seroprevalence among blood donors using a random effects model (serological diagnostic methods)

A random effects model analysis revealed a prevalence of *T. gondii *reactivity among blood donors, determined using serological diagnostic methods, at 35.7% (95% CI: 29.8-42%), accompanied by substantial heterogeneity between studies (I² = 99%, p < 0.001) (Figure 2). The influence diagnostics and the forest plot illustrating the results of the leave-one-out analysis are presented in Appendix D and Appendix E. According to them, no study was identified as influential.

When specifically looking at the prevalence of different types of antibodies, the estimates were as follows: 32% (95% CI: 26-38.2%) for IgG antibodies (I² = 99%, p < 0.001) (Appendix F), 0.8% (95% CI: 0.3-1.3%) for IgM antibodies (I² = 92%, p < 0.001) (Appendix G), and 1.2% (95% CI: 0.8-1.6%) for both IgG and IgM antibodies combined (I² = 81%, p < 0.001) (Appendix H). The influence diagnostics and the forest plot illustrating the results of the leave-one-out analysis are presented in Appendix I, Appendix J, Appendix K, Appendix L, Appendix M, and Appendix N. According to them, no study was identified as influential.

**Figure 3:**
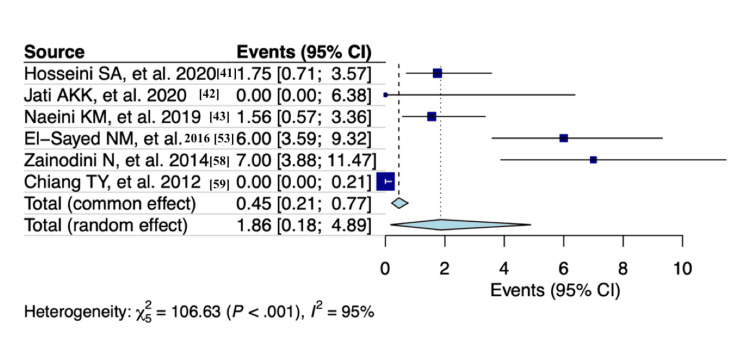
Forest plot evaluating the calculated prevalence of Toxoplasma gondii detection among blood donors using random effects model (molecular diagnostic procedures)

A random effects model analysis revealed a prevalence of *T. gondii *detection among blood donors, determined using molecular diagnostic methods, at 3.2% (95% CI: 0.4-8%), accompanied by substantial heterogeneity between studies (I² = 95%, p < 0.001). The influence diagnostics and the forest plot illustrating the results of the leave-one-out analysis are presented in Appendix O and Appendix P. According to them, the study conducted by Pawelczyk et al. (2022) [34] was identified as influential in this analysis. After the exclusion of the aforementioned study, the prevalence is estimated as 1.9% (95% CI: 0.2-4.9%) with substantial heterogeneity (I² = 95%, p < 0.001) (Figure 3).

Discussion

Our study highlights the prevalence of *T. gondii *reactivity and detection among blood donors, using a combination of serological and molecular diagnostic methods. While serological methods primarily measured the presence of *T. gondii *antibodies, molecular techniques provided an additional layer of detection by identifying the parasite’s DNA, thereby offering a comprehensive overview of *T. gondii *prevalence in this population.

Nonetheless, it is crucial to recognize that there is substantial heterogeneity among the research. There are multiple possible explanations for the substantial heterogeneity that was noted among the included studies. First, it is important to note that the current studies are observational and have been carried out under various circumstances, at various times, and in various locations. Consequently, variations in demographics and geographic areas under investigation likely contribute to the disparities in *T. gondii *reactivity and detection prevalence. Differences in prevalence and transmission severity are expected, as the disease is endemic in many regions.

Heterogeneity may also stem from differences in the age, sex, and health condition of blood donors, as well as variations in sample sizes, inclusion criteria, and diagnostic techniques. Disparities in the sensitivity, specificity, and accuracy of diagnostic methods (both molecular and serological) further contribute to these variations.

Our intention in presenting a pooled estimate is not to suggest uniformity across regions but to offer a global perspective that can inform broader public health strategies, especially in areas where local data are limited or unavailable. While local data are essential for tailoring interventions to specific regions, pooled estimates reveal overarching trends and provide a valuable benchmark for understanding the global burden of the disease.

It is crucial to consider these variables when analyzing prevalence estimates and addressing observed heterogeneity. Given the nature of this study, some variation in prevalence and incidence is anticipated. Therefore, in the context of proportional meta-analysis, a high I² does not necessarily imply inconsistency [25].

Foroutan-Rad et al. [64] conducted a meta-analysis that calculated the weighted overall prevalence of *T. gondii *antibodies to be 33% (95% CI: 28-39%) using serological methods. The seroprevalence of immunoglobulin IgM and both IgG and IgM antibodies was found to be 1.8% (95% CI: 1.1-2.4%) and 1.1% (95% CI: 0.3-1.8%), respectively. In another study, Wang et al. [65] calculated the prevalence of *T. gondii *infection in Chinese blood donors, reporting an overall IgG seroprevalence of 6.3% (95% CI: 4.6-8.1%). Lastly, the study conducted by Foroutan et al. [66] in 2024 found a moderate exposure rate to *T. gondii *among Iranian blood donors, with 32.9% seropositive for IgG, 1.4% for IgM, and 1.7% for both antibodies. Similar to the previous study, considerable heterogeneity was observed among the included studies.

Our findings align closely with those of previous meta-analyses, reaffirming the prevalence estimates of *T. gondii *reactivity among blood donors. While there is general agreement, minor variations were observed, which could be attributed to differences in the total number of studies included, study populations and geographical locations, database sources, different inclusion and exclusion criteria, quality assessments, analyses of outliers and influential analyses, inherent heterogeneity among the studies, and possible temporal changes in *T. gondii *reactivity prevalence. Besides this, one also has to consider the possible biases in sample selection for each study and different sensitivities and specificities of the diagnostic methods available that may further explain the prevalence differences observed between estimates.

In most cases, acute toxoplasmosis is self-limiting, and the majority of healthy adults without immunocompromisation or pregnancy do not necessitate treatment. Nevertheless, individuals with severe or prolonged symptoms and those showing signs of pneumonitis, myocarditis, meningoencephalitis, posterior uveitis, or polymyositis are advised to undergo treatment. The antimicrobial regimens employed for treating immunocompetent individuals are the same as those used for immunocompromised patients, which include either pyrimethamine combined with sulfadiazine and leucovorin or pyrimethamine combined with clindamycin and leucovorin [67,68].

Strengths and Limitations

The comprehensive methodology used for the literature search, study selection, inclusion/exclusion criteria, eligibility screening, quality assessment, and pooled analysis of prevalence data from 38 studies is a major strength of this investigation. However, caution is needed when interpreting the results due to significant, unidentified heterogeneity. The variability in outcomes across the included studies was expected, given the nature of this research [69,70]. Several factors likely contributed to this heterogeneity, including differences in diagnostic methods, sample sizes, inclusion criteria, and variations in age, sex, geographical location, contact with animals, soil exposure, dietary habits (such as consumption of unwashed vegetables or undercooked meat), income levels, and health status of blood donors, all of which may introduce bias in estimating the prevalence of *T. gondii*.

Additionally, a positive diagnostic result does not always indicate active infection, and variations in the sensitivity, specificity, and accuracy of diagnostic techniques (serological and molecular) can lead to discrepancies in detecting the parasite among blood donors. In this context, IgG and IgM results have distinct clinical and epidemiological implications. IgG antibodies reflect prior exposure and generally indicate long-term immunity; thus, a positive IgG result in a blood donor suggests past infection without necessarily indicating a current risk of transmission. Conversely, IgM antibodies are typically associated with recent or acute infection. However, the persistence of IgM for months or even years after infection complicates interpretation, as it may not reflect ongoing parasitemia. From a clinical perspective, IgM positivity may raise concern for potential transfusion-transmitted toxoplasmosis, especially if parasitemia is confirmed by molecular testing. From an epidemiological perspective, IgG prevalence indicates cumulative exposure within a population, while IgM prevalence can provide insight into recent transmission patterns. Therefore, separate reporting and interpretation of IgG and IgM are essential for accurate risk assessment and public health decision-making.

The regional heterogeneity in *T. gondii *reactivity and detection prevalence, testing practices, and study designs further limit the generalizability of the pooled international estimate to specific regions. While a global analysis offers a broad perspective, it may not fully reflect local epidemiological differences. Moreover, the persistence of IgM antibodies, which are typically indicative of acute infection but can remain in the bloodstream for extended periods, and the low sensitivity of PCR in blood complicate the accurate detection of *T. gondii*. These diagnostic limitations can affect the interpretation of prevalence rates in the included studies. Due to limited data (fewer than 10 studies reporting on variables such as male proportion, mean age, and comorbidities), these covariates were excluded from the analysis.

Additionally, passive antibodies from seropositive donors may transiently affect the immune status of recipients, potentially leading to misinterpretation of the recipient’s immunity. This meta-analysis was not registered in PROSPERO. Only observational studies published in English were included, resulting in further reporting bias. Given these considerations, it is important to acknowledge that, like any meta-analysis, our study is subject to potential biases, including selection bias, language bias, search bias, heterogeneity bias, confounding bias, and data extraction bias, all of which may influence the validity and generalizability of our findings.

## Conclusions

Our systematic review and meta-analysis on the prevalence of *T. gondii *reactivity and detection among blood donors highlight a significant public health issue. Enhanced screening protocols are crucial to protect vulnerable transfusion recipients. Rather than focusing solely on prevention, targeted screening can help detect active or recent infections and reduce transmission risk. Additionally, raising awareness about toxoplasmosis and promoting hygiene practices can contribute to risk reduction. These findings highlight the need for risk mitigation strategies and further localized research to improve transfusion safety.

## Figures and Tables

**Figure 4:**
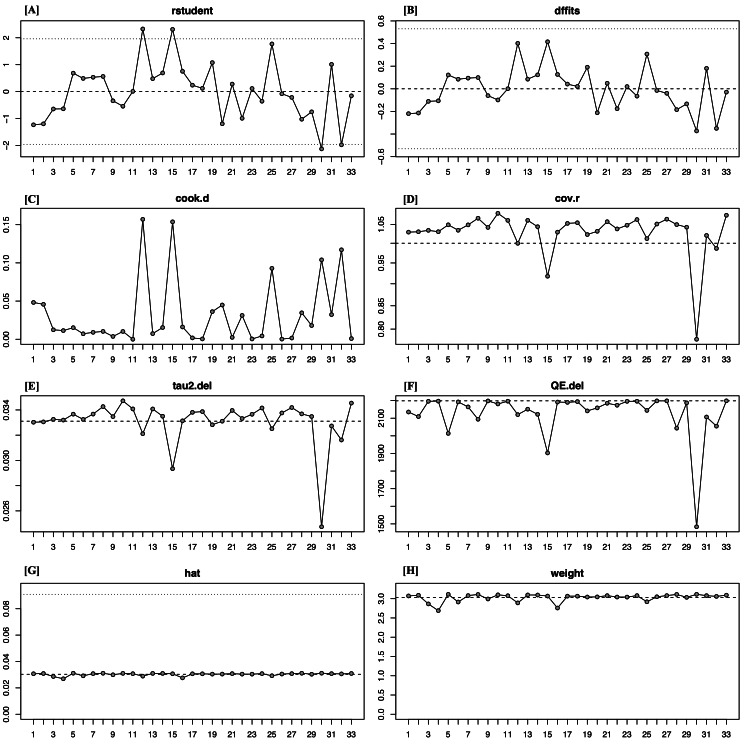
Graphical display of influence diagnostics for each included study assessing the prevalence of Toxoplasma gondii reactivity among blood donors (serological methods) (A) standardized residuals, (B) DFFITS, (C) Cook’s distances, (D) covariance ratios, (E) leave-one-out τ², (F) leave-one-out Q, (G) hat values, and (H) study weights

**Figure 5:**
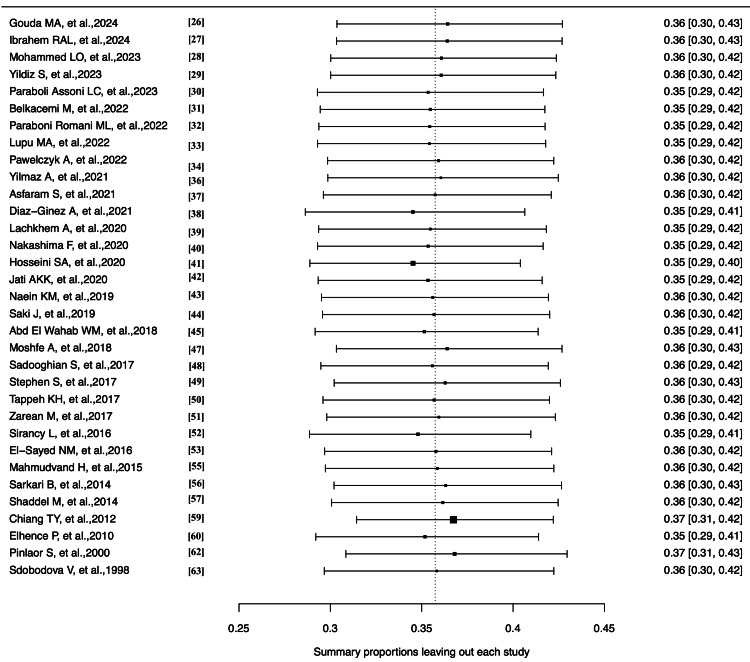
Forest plot showing the recalculated pooled estimates, each time excluding one study, based on the leave-one-out approach (serological methods)

**Figure 6:**
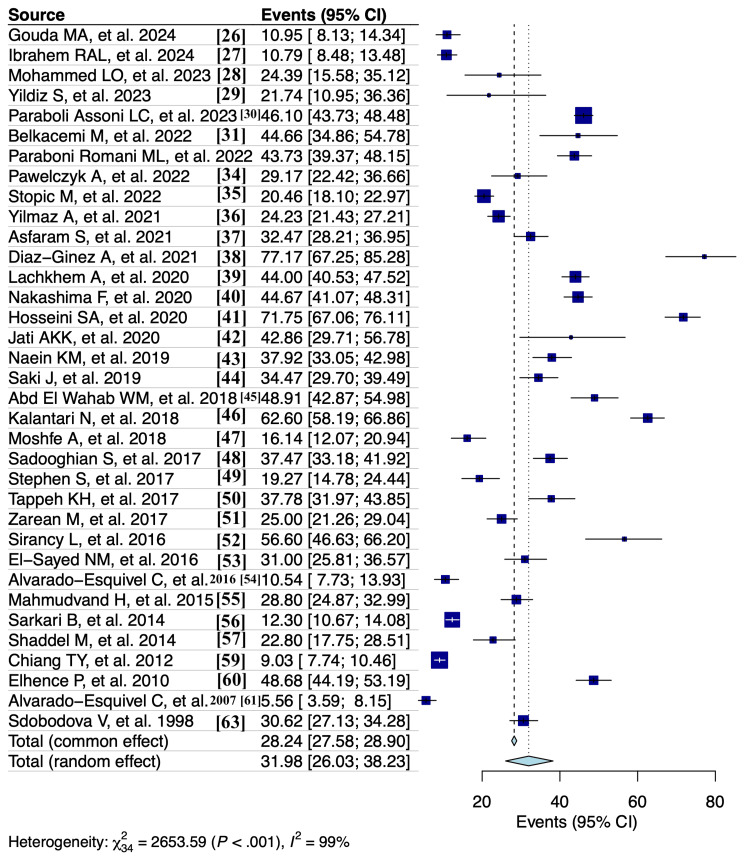
Forest plot showing Toxoplasma gondii seroprevalence among blood donors using a random effects model (IgG antibodies)

**Figure 7:**
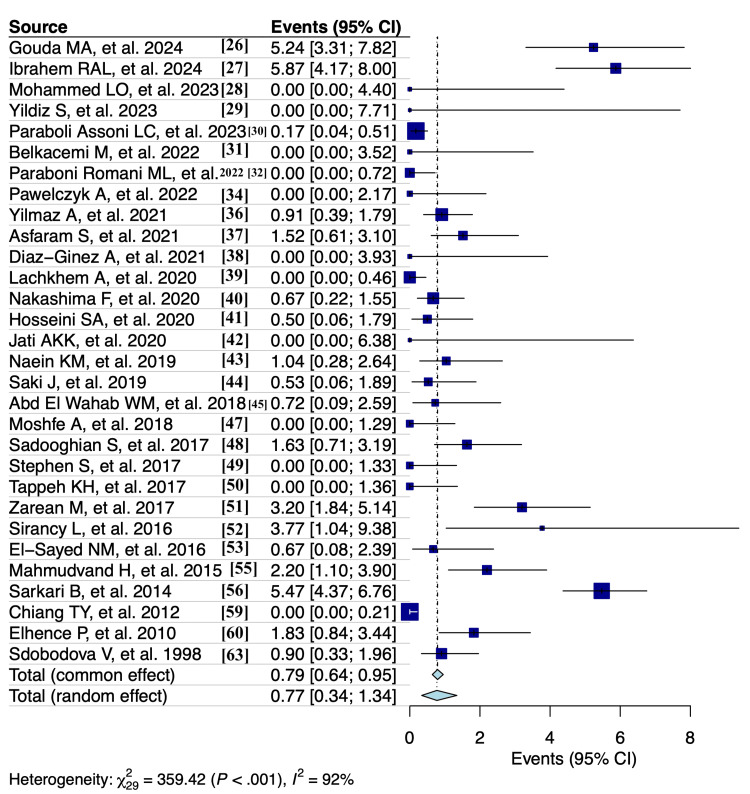
Forest plot showing Toxoplasma gondii seroprevalence among blood donors using a random effects model (IgM antibodies)

**Figure 8:**
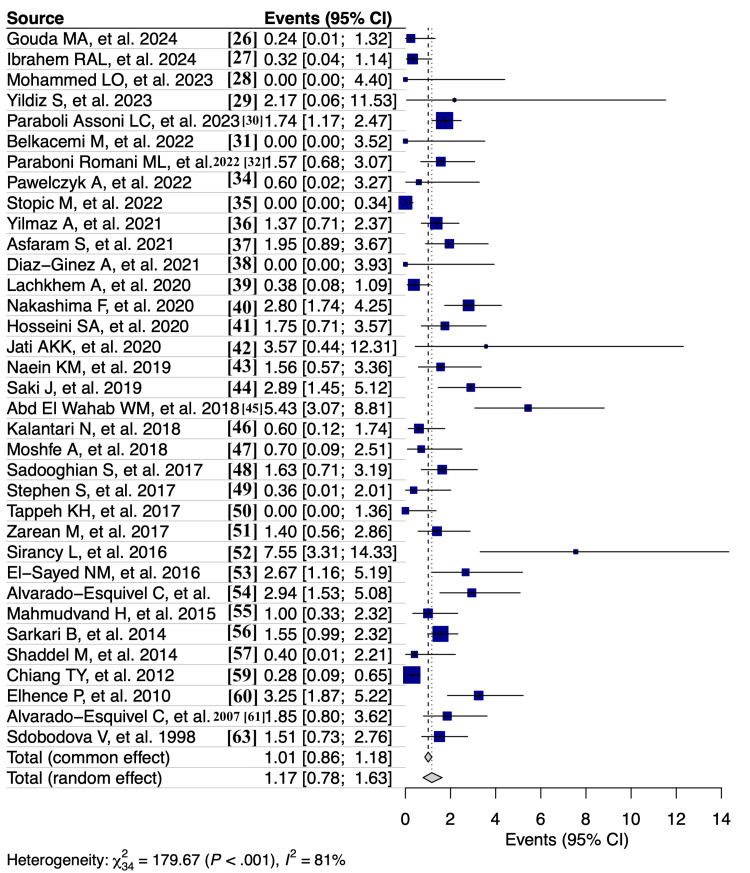
Forest plot showing Toxoplasma gondii seroprevalence among blood donors using a random effects model (both IgG and IgM antibodies)

**Figure 9:**
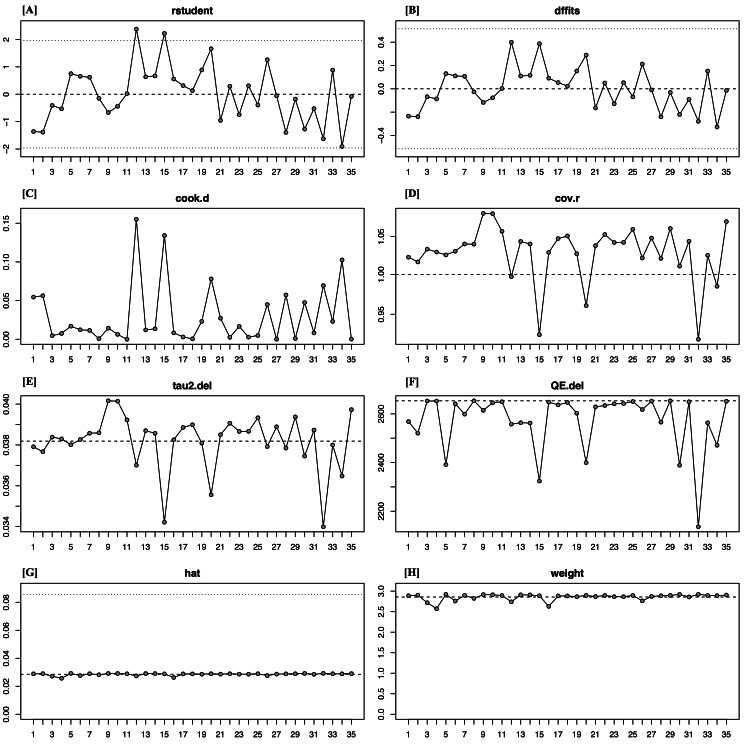
Graphical display of influence diagnostics for each included study assessing the prevalence of Toxoplasma gondii reactivity among blood donors (IgG antibodies) (A) standardized residuals, (B) DFFITS, (C) Cook’s distances, (D) covariance ratios, (E) leave-one-out τ², (F) leave-one-out Q, (G) hat values, and (H) study weights

**Figure 10:**
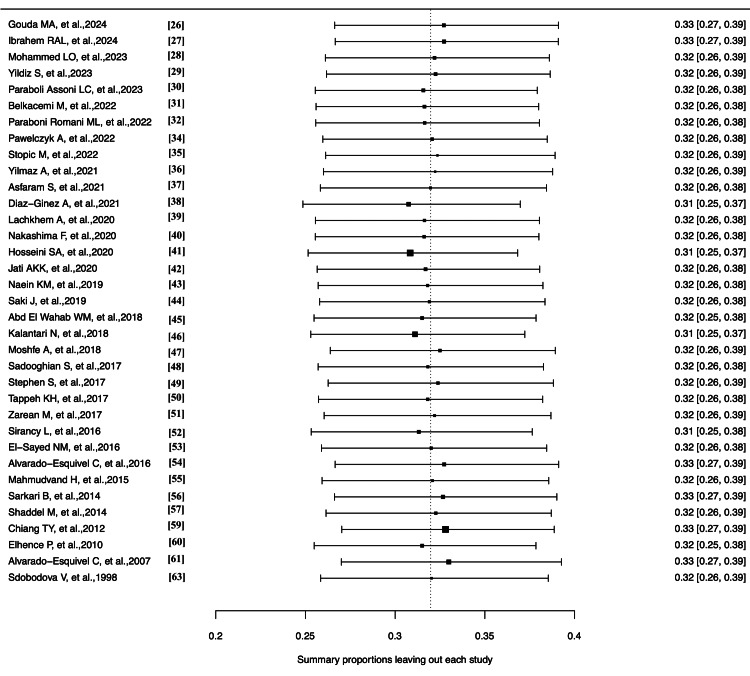
Forest plot showing the recalculated pooled estimates, each time excluding one study, based on the leave-one-out approach (IgG antibodies)

**Figure 11:**
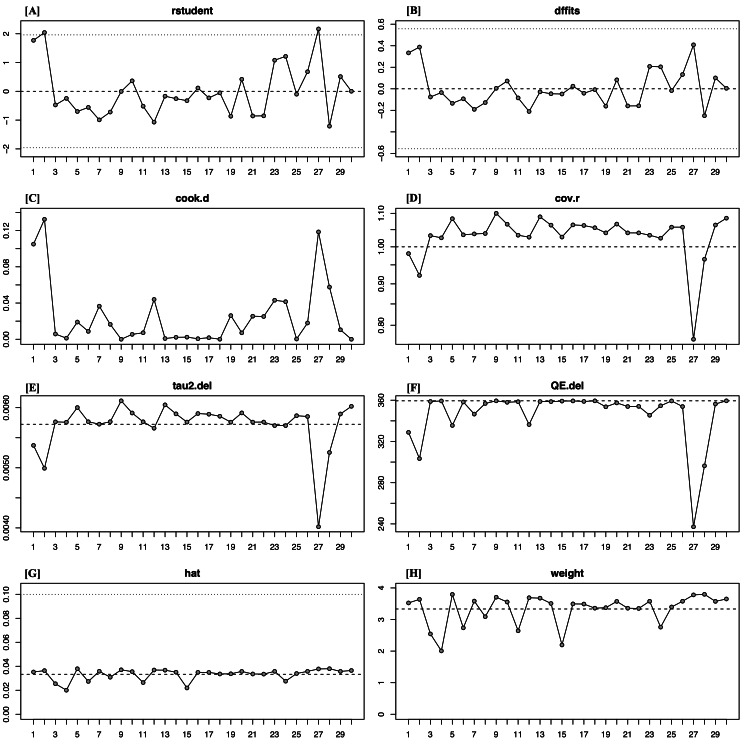
Graphical display of influence diagnostics for each included study assessing the prevalence of Toxoplasma gondii reactivity among blood donors (IgM antibodies) (A) standardized residuals, (B) DFFITS, (C) Cook’s distances, (D) covariance ratios, (E) leave-one-out τ², (F) leave-one-out Q, (G) hat values, and (H) study weights

**Figure 12:**
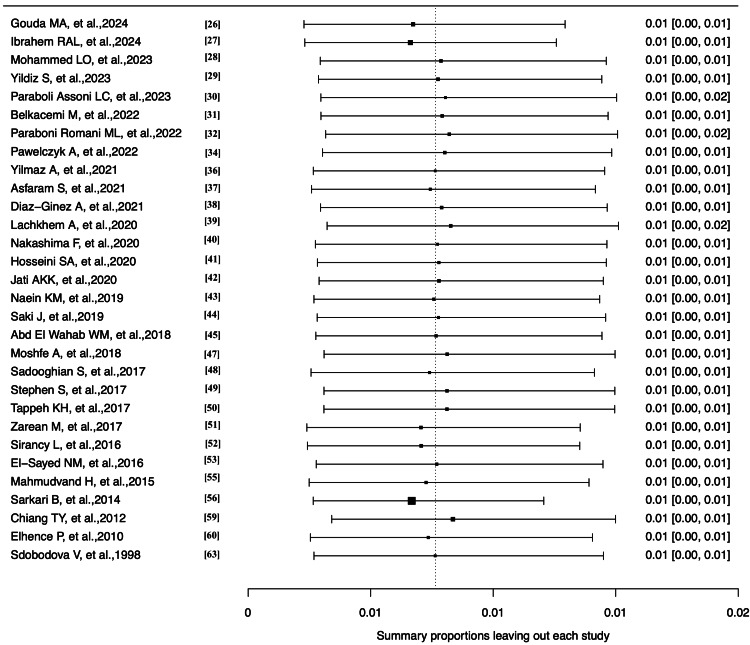
Forest plot showing the recalculated pooled estimates, each time excluding one study, based on the leave-one-out approach (IgM antibodies)

**Figure 13:**
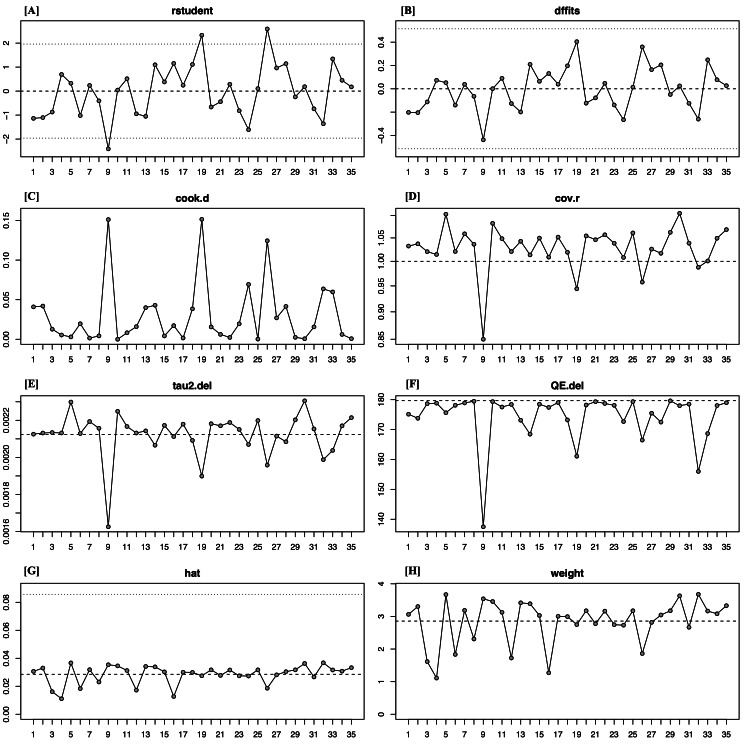
Graphical display of influence diagnostics for each included study assessing the prevalence of Toxoplasma gondii reactivity among blood donors (both IgG and IgM antibodies) (A) standardized residuals, (B) DFFITS, (C) Cook’s distances, (D) covariance ratios, (E) leave-one-out τ², (F) leave-one-out Q, (G) hat values, and (H) study weights

**Figure 14:**
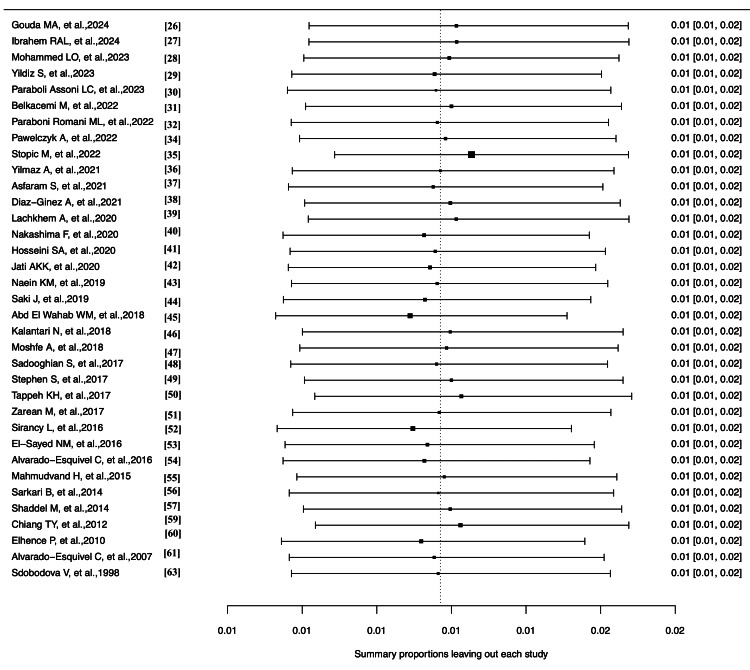
Forest plot showing the recalculated pooled estimates, each time excluding one study, based on the leave-one-out approach (both IgG and IgM antibodies)

**Figure 15:**
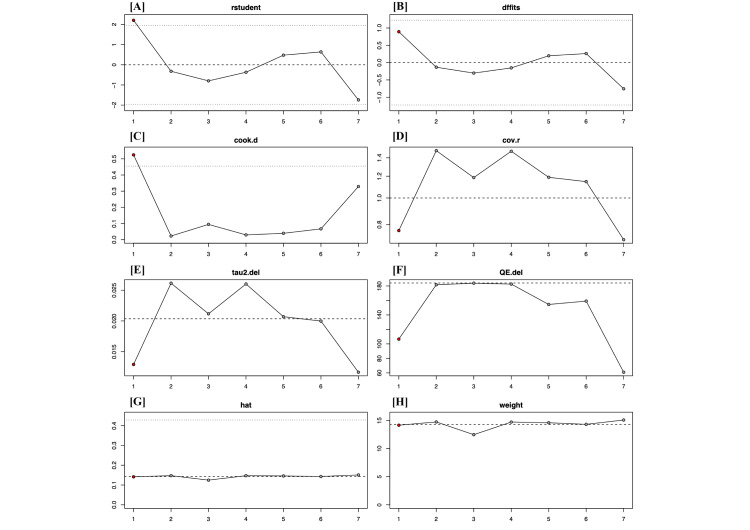
Graphical display of influence diagnostics for each included study assessing the prevalence of Toxoplasma gondii detection among blood donors (molecular diagnostic procedures)

**Figure 16:**
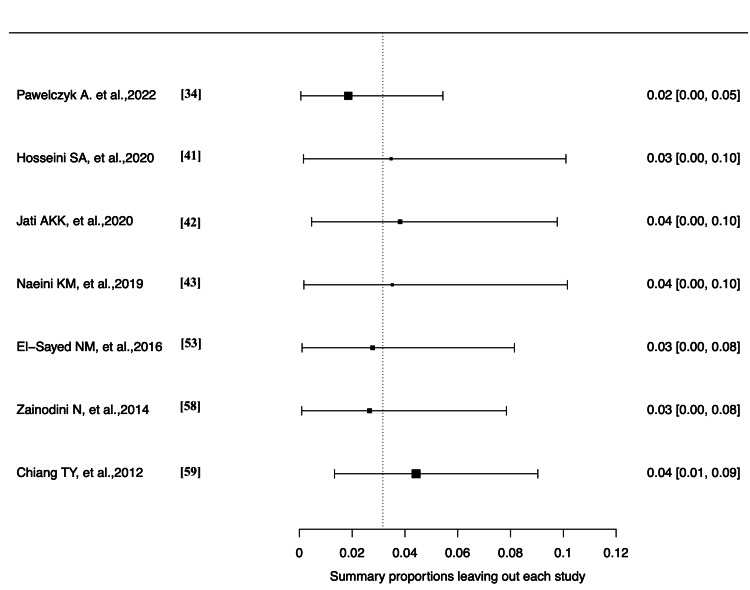
Forest plot showing the recalculated pooled estimates, each time excluding one study, based on the leave-one-out approach (molecular diagnostic procedures)

## Appendices

Appendix A 

**Table 4:** PRISMA checklist

Section and topic	Item #	Checklist item	Location where item is reported
TITLE			
Title	1	Identify the report as a systematic review.	Title
ABSTRACT			
Abstract	2	See the PRISMA 2020 for Abstracts checklist.	Abstract
INTRODUCTION			
Rationale	3	Describe the rationale for the review in the context of existing knowledge.	Introduction
Objectives	4	Provide an explicit statement of the objective(s) or question(s) the review addresses.	Introduction
METHODS			
Eligibility criteria	5	Specify the inclusion and exclusion criteria for the review and how studies were grouped for the syntheses.	Criteria for study selection and data extraction
Information sources	6	Specify all databases, registers, websites, organisations, reference lists and other sources searched or consulted to identify studies. Specify the date when each source was last searched or consulted.	Search strategy
Search strategy	7	Present the full search strategies for all databases, registers and websites, including any filters and limits used.	Search strategy
Selection process	8	Specify the methods used to decide whether a study met the inclusion criteria of the review, including how many reviewers screened each record and each report retrieved, whether they worked independently, and if applicable, details of automation tools used in the process.	Search strategy
Data collection process	9	Specify the methods used to collect data from reports, including how many reviewers collected data from each report, whether they worked independently, any processes for obtaining or confirming data from study investigators, and if applicable, details of automation tools used in the process.	Search strategy
Data items	10a	List and define all outcomes for which data were sought. Specify whether all results that were compatible with each outcome domain in each study were sought (e.g. for all measures, time points, analyses), and if not, the methods used to decide which results to collect.	Criteria for study selection and data extraction
	10b	List and define all other variables for which data were sought (e.g. participant and intervention characteristics, funding sources). Describe any assumptions made about any missing or unclear information.	Criteria for study selection and data extraction
Study risk of bias assessment	11	Specify the methods used to assess risk of bias in the included studies, including details of the tool(s) used, how many reviewers assessed each study and whether they worked independently, and if applicable, details of automation tools used in the process.	Quality assessment
Effect measures	12	Specify for each outcome the effect measure(s) (e.g. risk ratio, mean difference) used in the synthesis or presentation of results.	Statistical analysis
Synthesis methods	13a	Describe the processes used to decide which studies were eligible for each synthesis (e.g. tabulating the study intervention characteristics and comparing against the planned groups for each synthesis (item #5)).	Methods
	13b	Describe any methods required to prepare the data for presentation or synthesis, such as handling of missing summary statistics, or data conversions.	Statistical analysis
	13c	Describe any methods used to tabulate or visually display results of individual studies and syntheses.	Statistical analysis
	13d	Describe any methods used to synthesize results and provide a rationale for the choice(s). If meta-analysis was performed, describe the model(s), method(s) to identify the presence and extent of statistical heterogeneity, and software package(s) used.	Statistical analysis
	13e	Describe any methods used to explore possible causes of heterogeneity among study results (e.g. subgroup analysis, meta-regression).	Statistical analysis
	13f	Describe any sensitivity analyses conducted to assess robustness of the synthesized results.	Statistical analysis
Reporting bias assessment	14	Describe any methods used to assess risk of bias due to missing results in a synthesis (arising from reporting biases).	Methods
Certainty assessment	15	Describe any methods used to assess certainty (or confidence) in the body of evidence for an outcome.	Methods
RESULTS			
Study selection	16a	Describe the results of the search and selection process, from the number of records identified in the search to the number of studies included in the review, ideally using a flow diagram.	Methods, Figure 1
	16b	Cite studies that might appear to meet the inclusion criteria, but which were excluded, and explain why they were excluded.	Criteria for study selection and data extraction
Study characteristics	17	Cite each included study and present its characteristics.	Table 3
Risk of bias in studies	18	Present assessments of risk of bias for each included study.	Results
Results of individual studies	19	For all outcomes, present, for each study: (a) summary statistics for each group (where appropriate) and (b) an effect estimate and its precision (e.g. confidence/credible interval), ideally using structured tables or plots.	Prevalence of T. gondiireactivity and detection among blood donors
Results of syntheses	20a	For each synthesis, briefly summarise the characteristics and risk of bias among contributing studies.	Results
	20b	Present results of all statistical syntheses conducted. If meta-analysis was done, present for each the summary estimate and its precision (e.g. confidence/credible interval) and measures of statistical heterogeneity. If comparing groups, describe the direction of the effect.	Prevalence of T. gondiireactivity and detection among blood donors
	20c	Present results of all investigations of possible causes of heterogeneity among study results.	NA
	20d	Present results of all sensitivity analyses conducted to assess the robustness of the synthesized results.	Prevalence of T. gondiireactivity and detection among blood donors
Reporting biases	21	Present assessments of risk of bias due to missing results (arising from reporting biases) for each synthesis assessed.	Results
Certainty of evidence	22	Present assessments of certainty (or confidence) in the body of evidence for each outcome assessed.	Results
DISCUSSION			
Discussion	23a	Provide a general interpretation of the results in the context of other evidence.	Discussion
	23b	Discuss any limitations of the evidence included in the review.	Study’s strengths and limitations
	23c	Discuss any limitations of the review processes used.	Study’s strengths and limitations
	23d	Discuss implications of the results for practice, policy, and future research.	Discussion
OTHER INFORMATION			
Registration and protocol	24a	Provide registration information for the review, including register name and registration number, or state that the review was not registered.	NA
	24b	Indicate where the review protocol can be accessed, or state that a protocol was not prepared.	NA
	24c	Describe and explain any amendments to information provided at registration or in the protocol.	NA
Support	25	Describe sources of financial or non-financial support for the review, and the role of the funders or sponsors in the review.	Statements and Declarations
Competing interests	26	Declare any competing interests of review authors.	Statements and Declarations
Availability of data, code and other materials	27	Report which of the following are publicly available and where they can be found: template data collection forms; data extracted from included studies; data used for all analyses; analytic code; any other materials used in the review.	Availability of data and material

Appendix B 

**Table 5:** Literature search

Database	Search terms	Filters
Medline (Pubmed)	("toxoplasm*"[All Fields] OR "t.gondii"[All Fields]) AND ("blood"[MeSH Subheading] OR "blood"[All Fields] OR "blood"[MeSH Terms] OR "bloods"[All Fields] OR "haematology"[All Fields] OR "hematology"[MeSH Terms] OR "hematology"[All Fields] OR "haematoma"[All Fields] OR "hematoma"[MeSH Terms] OR "hematoma"[All Fields] OR "haemorrhage"[All Fields] OR "hemorrhage"[MeSH Terms] OR "hemorrhage"[All Fields] OR "haemorrhages"[All Fields] OR "hemorrhages"[All Fields] OR "haemorrhagic"[All Fields] OR "haemorrhaging"[All Fields] OR "hematologies"[All Fields] OR "haematomas"[All Fields] OR "hematomas"[All Fields] OR "hematoma s"[All Fields] OR "hematomae"[All Fields] OR "hemorrhaged"[All Fields] OR "hemorrhagic"[All Fields] OR "hemorrhagical"[All Fields] OR "hemorrhaging"[All Fields] OR ("haematological"[All Fields] OR "haematologically"[All Fields] OR "hematological"[All Fields] OR "hematologically"[All Fields] OR "hematology"[MeSH Terms] OR "hematology"[All Fields] OR "haematologic"[All Fields] OR "hematologic"[All Fields]) OR ("serum"[MeSH Terms] OR "serum"[All Fields] OR "serums"[All Fields] OR "serum s"[All Fields] OR "serumal"[All Fields]) OR ("plasma"[MeSH Terms] OR "plasma"[All Fields] OR "plasmas"[All Fields] OR "plasma s"[All Fields])) AND ("donor s"[All Fields] OR "tissue donors"[MeSH Terms] OR ("tissue"[All Fields] AND "donors"[All Fields]) OR "tissue donors"[All Fields] OR "donor"[All Fields] OR "donors"[All Fields] OR ("donor s"[All Fields] OR "tissue donors"[MeSH Terms] OR ("tissue"[All Fields] AND "donors"[All Fields]) OR "tissue donors"[All Fields] OR "donor"[All Fields] OR "donors"[All Fields]) OR ("donate"[All Fields] OR "donated"[All Fields] OR "donates"[All Fields] OR "donating"[All Fields] OR "donation"[All Fields] OR "donations"[All Fields] OR "donator"[All Fields] OR "donators"[All Fields]) OR "transfusi*"[All Fields])	-
Scopus	( toxoplasm* OR t.gondii ) AND ( blood OR hematologic OR serum OR plasma ) AND ( donor OR donors OR donation OR transfusi* )	-
Cochrane Central	( toxoplasm* OR t.gondii ) AND ( blood OR hematologic OR serum OR plasma ) AND ( donor OR donors OR donation OR transfusi* )	-

Appendix C 

**Table 6:** Quality assessment

Newcastle-Ottawa Scale	Selection	Comparability	Outcome
Gouda et al. [26]	***	*	*
Ibrahem et al. [27]	***	*	*
Mohammed et al. [28]	***	*	**
Yildiz et al. [29]	***	*	*
Assoni et al. [30]	**	*	*
Belkacemi et al. [31]	***	*	*
Paraboni et al. [32]	**	*	*
Lupu et al. [33]	**		**
Pawelczyk et al. [34]	***	*	*
Stopic et al. [35]	**	*	**
Yilmaz et al. [36]	**		**
Asfaram et al. [37]	**	*	*
Díaz-Ginéz and Silva-Díaz [38]	**	*	**
Lachkhem et al. [39]	***	*	*
Nakashima et al. [40]	***	*	**
Hosseini et al. [41]	***	*	**
Jati et al. [42]	***		**
Naeini et al. [43]	***	*	**
Saki J, et al. [44]	***	*	*
Abd El Wahab et al. [45]	***	*	**
Kalantari et al. [46]	***	*	*
Moshfe et al. [47]	***		*
Sadooghian et al. [48]	**	*	**
Stephen et al. [49]	**		**
Tappeh et al. [50]	***	*	*
Zarean et al. [51]	**		**
Siransy et al. [52]	**	*	**
El-Sayed et al. [53]	***	*	*
Alvarado-Esquivel et al. [54]	***	*	**
Mahmudvand et al. [55]	***	*	*
Sarkari et al. [56]	***		*
Shaddel et al. [57]	**	*	**
Zainodini et al. [58]	**		**
Chiang et al. [59]	***	*	*
Elhence et al. [60]	**		**
Alvarado-Esquivel et al. [61]	**	*	**
Pinlaor et al. [62]	***	*	*
Svobodová and Literák [63]	***	*	**

Appendix D 

Appendix E 

Appendix F 

Appendix G 

Appendix H 

Appendix I 

Appendix J 

Appendix K 

Appendix L 

Appendix M

Appendix N

Appendix O 

Appendix P
